# Detection and Characterization of Bacterial and Viral Acute Gastroenteritis among Outpatient Children under 5 Years Old in Guangzhou, China

**DOI:** 10.4269/ajtmh.23-0725

**Published:** 2024-02-27

**Authors:** Xin Luo, Jiankai Deng, Mingyong Luo, Nan Yu, Xiaoyan Che

**Affiliations:** ^1^ Department of Laboratory Medicine, Guangdong Women and Children Hospital, Guangzhou, China;; ^2^ Department of Laboratory Medicine, The First Affiliated Hospital, Sun Yat-sen University, Guangzhou, China;; ^3^ Laboratory of Emerging Infectious Diseases and Division of Laboratory Medicine, Zhujiang Hospital, Southern Medical University, Guangzhou, China

## Abstract

Acute gastroenteritis (AGE) in children can be attributed to a multitude of bacterial and viral pathogens. The objective of this study was to investigate the epidemiology of bacterial and viral AGE in children and to compare clinical characteristics between single and multiple enteric pathogen infections. A total of 456 stool samples were collected from outpatient children under 5 years old with AGE, which were subsequently analyzed for nine bacteria and three viruses using the Luminex xTAG^®^ Gastrointestinal Pathogen Panel. The presence of at least one pathogen was detected in 260 cases (57.0%), with *Salmonella* being the predominant agent, followed by norovirus, *Campylobacter*, and rotavirus. A total of 69 cases (15.1%) exhibited positive results for two or more enteric pathogens. Although certain co-infections demonstrated significant differences in primary clinical features compared with mono-infections, no statistical variance was observed in terms of disease severity. In outpatient children from southern China, *Salmonella* emerged as the most prevalent causative agent of AGE, succeeded by norovirus and *Campylobacter.* This study underscores the burden posed by coinfections and highlights the clinical characteristics associated with AGE when accompanied by coinfections among children under 5 years old.

## INTRODUCTION

Acute gastroenteritis (AGE), which affects billions of individuals globally annually, ranks as the fifth leading cause of mortality in children under 5 years old, resulting in approximately 2.5 million deaths each year.^1^^–^^3^ It poses a continuous threat to public health, particularly among vulnerable populations such as young children, older adults, malnourished individuals, and those with compromised immune systems.^4^^–^^6^

A variety of pathogens, including rotavirus, norovirus, *Salmonella*, and *Campylobacter*, have been demonstrated to be causative agents of AGE.^2^ In an etiological study conducted on children with AGE in Korea from 2004 to 2019, *Escherichia coli* and *Salmonella* were identified as the most prevalent bacterial pathogens, followed by *Campylobacter*.^7^ The incidence of norovirus infections exhibited a gradual increase and accounted for the majority of viral AGE cases.^7^ In a recent epidemiological study conducted in Japan, viral AGE was found to be associated with multiple virus infections in 33.1% of cases, with norovirus being the predominant pathogen involved.^8^ Despite the availability of rotavirus vaccination since 2001 in China, it has not been included in the national immunization program. Luo et al.^9^ reported that rotavirus remained the primary viral pathogen followed by norovirus among outpatient children with AGE in Guangzhou, China. However, comprehensive investigations encompassing a wide range of pathogens including both bacteria and viruses are rarely reported for AGE patients. Furthermore, there is limited understanding regarding clinical symptoms and coinfection patterns of bacteria and viruses due to constraints imposed by detection methodologies.

This investigation aims to explore the epidemiology of bacterial and viral AGE in outpatients, and to compare clinical characteristics of AGE in children under 5 years old with single versus multiple enteric pathogen infections in China.

## MATERIALS AND METHODS

### Sample collection.

From January 2014 to December 2015, fecal specimens were prospectively collected from outpatient children under age 5 years diagnosed with AGE at our hospital in Guangzhou, China. Patients who did not provide fecal specimens for laboratory analysis were excluded from the study. The diagnostic criteria for AGE are sudden onset of diarrhea (>3 times) or vomiting (>3 times) in the preceding 24 hours with symptoms lasting no longer than 7 days. Informed consent was obtained from the patients or their parents before fecal specimen collection. Demographic and clinical information of outpatient cases with AGE were acquired through paper-based questionnaires. Laboratory findings in stool, including occult blood (OB) test and white blood cell (WBC) count, were received from patients’ clinical database.

The fresh fecal specimens received in our laboratory, each exceeding 100 *µ*L/150 mg, were promptly stored at –70°C for subsequent investigation. Collected fecal specimens were processed using a NucliSENS easyMAG Lysis Buffer (BioMérieux, Marcy-l’Étoile, France), followed by nucleic acid isolation using the QIAamp MinElute Virus Spin kit (Qiagen, Hilden, Germany). The xTAG Gastrointestinal Pathogen Panel assay (xTAG GPP) was conducted according to the manufacturer’s instructions to detect nine bacteria and three viruses, including *Salmonella* spp., *Shigella* spp., *Campylobacter* spp., *Yersinia enterocolitica*, *Vibrio cholerae, Escherichia coli* O157 (*E*. *coli* O157), enterotoxigenic *E. coli* (ETEC) LT/ST, Shiga-like toxin-producing *E. coli* (STEC) stx1/stx2, *Clostridium difficile* toxins A/B, rotavirus A, adenovirus 40/41, and norovirus GI/GII.

## STATISTICAL ANALYSES

The statistical analysis was conducted using SPSS software (version 21.0, IBM SPSS, Armonk, NY). Comparisons between groups were performed using the chi-square test, Fisher exact test, or Mann–Whitney *U* test. A *P*-value <0.05 was considered statistically significant.

## RESULTS

### Subjects and overall detection.

A total of 456 outpatient children under 5 years (61.4% male) participated in this investigation, with a median age of 14 months. The xTAG GPP assay results revealed that 57.0% (260/456) of the stool specimens tested positive for one or more enteric pathogens, resulting in a total of 335 pathogens (204 bacterial strains and 131 viral strains). *Salmonella* accounted for the largest proportion (16.9%, 77/456), followed by norovirus (15.4%, 70/456), *Campylobacter* (12.5%, 57/456), rotavirus (12.3%, 56/456), and *C. difficile* (10.1%, 46/456). Other enteric pathogens detected, in descending order, included ETEC (2.6%, 12/456), *E. coli* O157 (1.3%, 6/456), adenovirus 40/41 (1.1%, 5/456), *Y. enterocolitica* (0.7%, 3/456), STEC (0.4%, 2/456), and *Shigella* (0.2%, 1/456). *Vibrio cholerae* was not detected (Table 1).

**Table 1:** Distribution of detected pathogens in children with acute gastroenteritis

Pathogens	Total, *N*	Single Infection, *n* (%)	Coinfection, *n* (%)
Bacteria	204	106	98
*Salmonella*	77	42 (54.55)	35 (45.45)
*Campylobacter*	57	36 (63.16)	21 (36.84)
*Clostridium difficile* (toxins A/B)	46	21 (45.65)	25 (54.35)
ETEC	12	3 (25.00)	9 (75.00)
*E. coli* O157	6	1 (16.67)	5 (83.33)
STEC	2	1 (50.00)	1 (50.00)
*Yersinia enterocolitica*	3	1 (33.33)	2 (66.67)
Shigella	1	1 (100.00)	0 (0.00)
Virus	131	85	46
Rotavirus A	56	38 (67.86)	18 (32.14)
Norovirus	70	45 (64.29)	25 (35.71)
Enteric adenovirus 40/41	5	2 (40.00)	3 (60.00)

ETEC = enterotoxigenic *Escherichia coli*; *E. coli* O157 = *Escherichia coli* O157; STEC = Shiga-like toxin-producing *Escherichia coli.*

We also observed that 15.1% (69/456) of the fecal specimens exhibited positivity for a minimum of two pathogens. Among these children with AGE, coinfections involving bacteria and viruses were identified in 35 cases, followed by multiple bacterial infections and multiple viral infections in 29 and five cases, respectively.

### Seasonal and age distribution of enteric pathogens in children with AGE.

**Figure 1.**
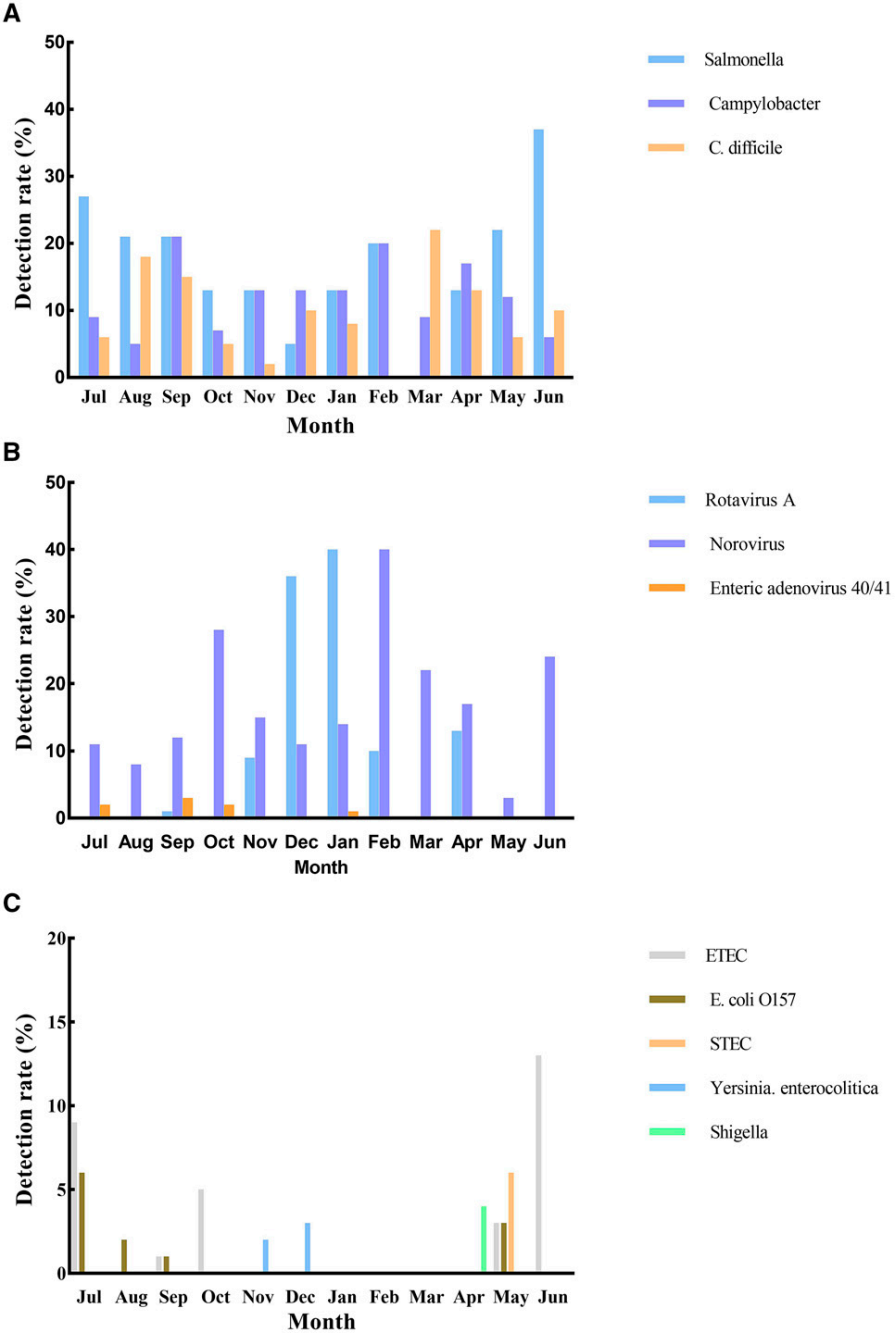
Monthly distribution of pathogens in children with acute gastroenteritis. (**A**) *Salmonella*, *Campylobacter*, and *Clostridium difficile*. (**B**) Rotavirus A, norovirus, and enteric adenovirus40/4. (**C**) enterotoxigenic *Escherichia coli* (ETEC), *E*. *coli* O157, Shiga-like toxin-producing *E. coli* (STEC), *Yersinia enterocolitica*, and *Shigella.*

The seasonal distribution of enteric pathogens in children with AGE is presented in Figure 1. *Salmonella* and ETEC were predominantly detected during the months of June and July. Rotavirus exhibited its peak occurrence in January, whereas norovirus peaked in February. *Campylobacter* and norovirus were observed throughout the year. No notable seasonal patterns could be discerned for other enteric pathogens.

**Figure 2.**
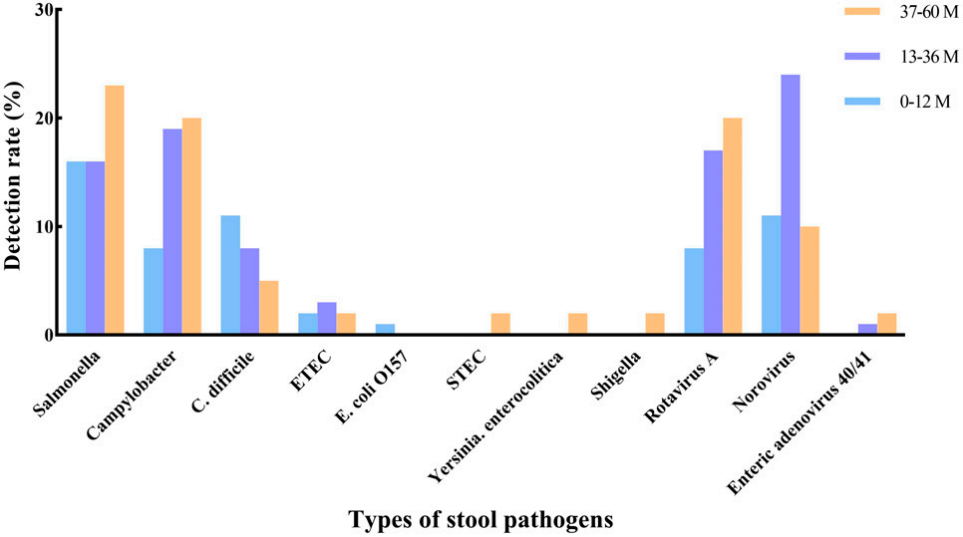
Age distribution of pathogens in children with acute gastroenteritis.

Figure 2 illustrates the age distribution of enteric pathogens in children with AGE. *Salmonella*, *Campylobacter*, and rotavirus were mostly found in older children (37–60 mouths), whereas norovirus had the highest detection rate among those aged 13–36 months.

Among the 46 cases of *C. difficile* infection, 33 (71.7%) occurred in infants younger than 12 months, accounting for 11% of all cases at this age group. *Yersinia enterocolitica*, STEC, and *Shigella* were only observed in older children (37–60 mouths), whereas *E. coli* O157 was more commonly found in younger children (0–12 months). Notably, there was no significant difference in the median age of children with AGE between single-pathogen infections and coinfections, as shown in Table 2.

**Table 2:** Comparison of clinical characteristics in the bacterial and viral infection among children with acute gastroenteritis in Guangzhou, China, 2014–2015

Variables	Single Bacterial Infection (*n =* 106)	Single Viral Infection (*n =* 85)	Coinfection (*n =* 69)		
			Viral–Bacterial (*n =* 35)	Multiple Bacterial (*n =* 29)	Multiple Viral (*n =* 5)
Demographics					
Male, *n* (%)*	59 (55.66)	54 (63.53)	23 (65.71)	19 (65.52)	4 (80.00)
Age range (month)	1–60	0.56–60	3–60	1–53	11–35
Median age in months (IQR)*	12 (8.00–22.50)	13 (10.00–23.00)	14 (11.00–25.00)	11 (7.50–17.50)	19 (11.50–29.50)
Clinical presentations					
Fever >37.5 °C, *n* (%)*	30 (28.30)	25 (29.41)	9 (25.71)	8 (27.59)	1 (20.00)
Vomit, *n* (%)	9 (8.49)^†^	40 (47.06)	13 (37.14)	5 (17.24)	3 (60.00)
Diarrhea, *n* (%)	106 (100.00)	85 (100.00)	35 (100.00)	29 (100.00)	5 (100.00)
Frequency (times/day), median (IQR)*	5 (4.00–6.00)	5 (4.00–7.00)	5 (3.00–8.00)	5.5 (4.00–8.00)	5 (3.00–5.50)
Laboratory findings in stool					
WBC (/HP), *n* (%)	20 (18.87)	1 (1.18)^‡^	8 (22.86)	5 (17.24)	0 (0.00)
OB positive, *n* (%)	58 (54.72)^§^	13 (15.29)	9 (25.71)	9 (31.03)	0 (0.00)
Vesikari score*	4 (3–5)	5 (4–7)	4 (2–7)	5 (3–6)	5 (3–8)

HP = high power field; IQR = interquartile range; OB = stool occult blood test; WBC = white blood cell count.

* *P* >0.05, single bacterial infection compared with single viral infection, viral–bacterial coinfection, and multiple bacterial coinfection; single viral infection compared with viral–bacterial coinfection and multiple viral coinfection.

^†^ *P* <0.05, compared with single viral infection and viral-bacterial coinfection.

^‡^ *P* <0.05, compared with single bacterial infection and viral–bacterial coinfection.

^§^ *P* <0.05, compared with single viral infection, viral–bacterial coinfection, and multiple bacterial coinfection.

### Clinical features of enteric pathogen infections in children with AGE.

Pairwise comparisons were conducted to assess the clinical features of single bacteria/virus infections and coinfections involving multiple pathogens (Table 2). Similar to single bacterial infections versus single viral infections (*P* <0.0001), significant differences in vomiting were also observed between single bacterial infections and viral–bacterial coinfections (*P* <0.0001). Single bacterial infections exhibited a higher likelihood of obtaining a positive result on the OB test compared with single viral infections (*P* <0.0001), viral–bacterial coinfections (*P* = 0.0034), and multiple bacterial coinfections (*P* = 0.0351). Significant differences in WBC count were found between two groups, including single viral infections versus single bacterial infections (*P* <0.0001) and single viral infections versus viral-bacterial coinfections (*P* = 0.0002). However, no significant differences in the clinical characteristics such as gender, age, fever, or diarrhea frequency were observed among cases of single bacterial/viral infections and coinfections of viral–bacterial, multiple bacteria and multiple virus. The clinical Vesikari score was used to evaluate the severity of diseases, and the median Vesikari score for these five groups ranged from 4 to 5. There was no statistically significant difference in disease severity between AGE children with single bacterial/viral infections and those exhibiting coinfections.

## DISCUSSION

As one of the most prevalent global illnesses, AGE has garnered significant attention from researchers. Although numerous epidemiological studies have primarily focused on viral and bacterial causes of AGE, our understanding regarding the impact of a diverse range of pathogens remains limited.^1^^–^^3^^,^^7^^,^^8^ Therefore, we conducted a comprehensive investigation in Guangzhou, China, to elucidate the role of viruses and bacteria in this disease.

We could detect pathogens in 57% of AGE children. Consistent with our findings, de Wit et al.^10^ reported pathogen detection in approximately 40% of the AGE patients. The present study’s detection rate of viral pathogens was 28.7%, which agrees with an early report demonstrating that 20.0% to 30.0% of AGE patients were infected with the virus.^11^ It is well documented that norovirus and rotavirus are the two major viral pathogens associated with AGE worldwide.^12^ Rotavirus accounted for 12.3% of all AGE outpatients in our study, consistent with previous studies.^13^^–^^15^ Despite a significant reduction in rotavirus infections due to vaccine introduction and utilization, norovirus has emerged as the predominant cause of viral AGE.^16^^–^^19^ In our study, norovirus was the most common detected viral pathogen, accounting for 15.4% of AGE patients, which aligns with findings from Huzhou (20.2%), the United States (18.5%), Czech Republic (11.8%), and Indonesia (12.3%).^20^^–^^23^ In the sentinel study of Qu et al.,^24^ diarrheagenic *E. coli* was detected more frequently, followed by *Salmonella* and *Shigella*. Another study of de Wit et al.^10^ showed that *Campylobacter* was the most common pathogen, followed by rotavirus, norovirus, and *Salmonella*. In contrast to the foregoing ranking, our study found that *Salmonella* (16.9%) was more prevalent than other bacterial pathogens, such as *Campylobacter* (12.5%), *C. difficile* (10.1%), and other gut bacteria, which aligns with previous studies in Taiwan and Korea.^11^^,^^25^ Taken together, *Salmonella* and norovirus were major pathogens in outpatient children under 5 years with AGE, followed by *Campylobacter* and rotavirus in Guangzhou, China.

In our study, the incidence of rotavirus peaked in January, and norovirus was most commonly detected in February. Our study confirmed the previous findings that most AGE cases occurring during the winter months (December–February) are commonly associated with norovirus or rotavirus.^26^^,^^27^ We also found that *Salmonella* had a higher detection rate in June and July, which was supported by results from Taiwan.^25^ Most *Salmonella* and *Campylobacter* positive cases were in children aged 37–60 months, which was in line with the report that the proportion of all causative bacteria was highest in children aged 3–5 years.^24^ Due to the low detection rates, *Y. enterocolitica*, STEC, *Shigella*, *E. coli* O157, and adenovirus showed no variation with age.

Notably, 71.7% of *C. difficile*–positive detections in this study were observed in children younger than 12 months, and more than half of the outpatients with *C. difficile* positivity had one or more co-detected pathogens. Hung et al.^28^ reported that asymptomatic colonization of *C. difficile* is prevalent among infants. However, accumulating evidence suggests that 4.8% to 19.8% of AGE cases in infants and young children are associated with *C. difficile*.^17^^,^^29^^–^^31^ These findings underscore the need for further investigation into recent antibiotic use, chemotherapy treatment, or hospitalization as potential factors contributing to AGE caused by *C. difficile* infection. In general, a comprehensive understanding of the etiology and epidemiology of AGE in children warrants continuous monitoring due to regional variations in pathogen prevalence.

Although numerous studies have reported on bacteria- or virus-related AGE in outpatients worldwide, there is a paucity of clinical data comparing the features of multiple pathogen infections to those caused by single pathogens. The current study revealed a coinfection rate of 15.1% among children with AGE, which is consistent with the range of 10% to 17% reported in previous studies.^25^^,^^32^^,^^33^ Notably, our findings highlight norovirus and *Salmonella* as the most frequently detected pathogens responsible for coinfection, corroborated by data from other research groups.^25^^,^^32^^,^^33^ Pairwise comparisons were conducted to assess clinical features between single bacterial/viral infections and coinfections by multiple pathogens in this study. Our findings suggest that bacterial infections are primarily associated with blood and white blood cells in the stool, whereas vomiting is frequently observed in cases of viral infections, consistent with previous research.^34^^,^^35^

Researchers in China and elsewhere have reached divergent conclusions regarding whether coinfection contributes to more severe clinical symptoms.^34^^–^^36^ Our data demonstrate that there is no statistically significant difference in disease severity between children with AGE who have single bacterial/viral infections and those with coinfections. However, determining the specific pathogen responsible for the clinical symptoms in cases of multiple pathogen infections remains a challenge. The interactions among multiple pathogens within the human intestinal system are still poorly understood. It is reasonable to speculate that coinfection is relatively common in children with AGE; however, interpreting the detection of multiple targets on a multiplex panel in a clinical context poses uncertainties.^37^ Therefore, further investigations into pathogen load and underlying mechanisms are warranted.

The limitations of our study include the inclusion of data from only a 2-year period. Overall detection rates could have been enhanced by incorporating astrovirus and sapovirus, which were not targeted pathogens in the xTAG GPP assay. Furthermore, none of the 196 fecal specimens yielded positive pathogen detection; however, alternative enteropathogen multiplex nucleic acid amplification tests capable of detecting various bacteria and viruses may have provided positive results.

In conclusion, our data unequivocally substantiate *Salmonella* and norovirus as the predominant pathogens accountable for AGE in outpatient children aged younger than 5 years in Guangzhou, China. Furthermore, this comprehensive dataset significantly contributes to enhancing our understanding of the clinical manifestation and disease severity associated with co-infecting pathogens.
